# Study on the Causes, Types, and Mechanisms of Childhood Injuries-Age and Disease Specificity

**DOI:** 10.31662/jmaj.2019-0050

**Published:** 2021-07-09

**Authors:** Nobuo Matsuura, Yoshifumi Nishida, Shohei Harada, Kaoru Takahashi, Kazue Koshikawa, Shinya Konn, Nozomi Hosoda, Kimiko Deguchi, Utae Hotta, Toshiaki Oka

**Affiliations:** 1 Department of Early Childhood Education, Seitoku University, Matsudo, Japan; 2 National Institute of Advanced Industrial Science and Technology, Tokyo, Japan; 3 Department of Pediatrics, Kitasato University, Sagamihara, Japan; 4 Sagamihara-Ryouikuenn, Sagamihara, Japan; 5 Deguchi Pediatric Clinic, Ohmura, Japan; 6 Seitoku Primary School, Matsudo, Japan; 7 Pediatric Clinic, Sapporo Tokushukai Hospital, Sapporo, Japan

**Keywords:** Childhood injury, Nursery school and kindergarten, cause of injury, type of injury, Bodygraphic Injury Surveillance System (BISS)

## Abstract

**Introduction:**

To clarify the causes, types, and mechanisms of injuries in children, we collected injury cases and analyzed their causes.

**Methods:**

During the 3-year period from 2013, we collected injury cases from three sources: nursery schools and kindergartens (A), emergency clinics of hospitals (B), and schools and a clinic for the developmentally disabled (C), using a format designed by Safe Kids Japan.

**Results:**

In all, 383 cases were collected during the 3-year period. The causes of the injuries in group A were crashes, falls, and so on. The types of injuries were cuts, bruises, fractures, injuries of teeth, etc. Dislocations and abrasions were prominent in nursery school children (aged less than 3 years) and bone fractures were prominent in kindergarten children aged more than 3 years.

Group B consisted of 144 cases. The most common causes of injuries were falls, traffic accidents, and so on, and the types of injuries were fractures, abrasions, sprains, etc. The incidence of fractures was particularly high and 50% of the accidents were bicycle accidents.

Group C consisted of 41 cases. Although the age distribution was similar to that of group B, the types of accidents and injuries were similar to those of group A.

The Bodygraphic Injury Surveillance System (BISS) analysis showed that groups A and C were similar, that is, injuries occurred mainly to the head, whereas in group B, the extremities were mainly affected.

**Conclusions:**

We analyzed the causes, types, and mechanisms of childhood injuries. The BISS may help to clarify the mechanisms of injuries in childhood.

## Introduction

Childhood injuries as a result of accidents are common in children and are the main causes of death in all pediatric age groups ^(1), (2), (3)^. The term “accident” has long been used in our country to mean unavoidable injuries due to the carelessness of parents or teachers of kindergartens or nursery schools. Recently, the term “injuries” instead of accidents has been used in North American and European countries ^(4)^. Injuries could be preventable if their mechanisms and causes are clarified and measures for their prevention are established ^(5)^.

Seitoku University is one of the largest universities in Japan for producing specialists in early childhood education, such as nursery school, kindergarten, and primary school teachers. Education for the prevention of injuries is one of the most important subjects in this field. The Ministry of Education, Culture, Sports, Science and Technology and the Ministry of Health, Labour and Welfare of Japan have made manuals for the prevention of injuries, as have nursery schools and kindergartens. However, the incidence of injuries has not decreased from 1960 ^(5)^.

Safe Kids Worldwide, organized for the prevention of childhood injuries, was established in the United States in 1988 ^(6)^. Safe Kids Japan, the CEO of which is Dr. Tatsuhiro Yamanaka, was organized and started activities to prevent injuries in our country in 2014 ^(7)^. According to the American Academy of Pediatrics, pediatricians have a responsibility to help prevent childhood injuries ^(1)^. The Japan Pediatric Society (JPS) Committee of Pediatric Emergency Medicine also acts to prevent childhood injuries ^(8)^. Case reports collected by members of this committee of the JPS are published in the society’s journal ^(8)^.

The Childhood Injury Prevention Engineering Council (CIPEC) of the Ministry of Economy, Trade and Industry was established in 1988 to support activities for the prevention and analysis of injuries. The CIPEC collects data on injuries from medical facilities, emergency clinics, administrators, fire departments, the police, schools, etc., and analyzes the mechanisms of injuries to establish models for their prevention ^(9)^. The systemic collection of injury cases has been performed in the cities of Oomura ^(10)^, Kyoto ^(11)^, and Sapporo ^(12)^.

Childhood injuries occur all over the world; however, the causes and types of unintentional childhood injuries differ in industrialized countries ^(13), (14)^, between indigenous and nonindigenous children ^(15)^, and between urban and suburban areas ^(16)^.

In this paper, we report our study of injury cases from multiple institutions and analyze the backgrounds of the injuries using the Bodygraphic Injury Surveillance System (BISS) ^(17)^.

## Materials and Methods

We collected injury cases from three sources: nursery schools and kindergartens (group A); emergency clinics of general hospitals and university hospitals, including the nurse’s room in primary schools (group B); and a nurse’s room and schools for handicapped children (group C). Questionnaires for this project were sent to all nursery schools and kindergartens in the Kanto area where students of Seitoku University and educational institutes practice child care for handicapped children in Chiba prefecture.

The questionnaire used was the same as that used by Safe Kids Japan at the National Center for Child Health and Development and inquired about name, birth date, sex, when and how the injuries occurred, and the dominant hand. The levels of injuries were those determined by the clinic or hospital at which diagnosis and treatment occurred. When the questionnaires were returned to the secretariat of Seitoku University, the data were input into a computer and saved on a CD-ROM that was sent to the National Institute of Advanced Industrial Science and Technology (AIST) for analyses using the BISS ^(17)^. The BISS is a new system developed by the AIST that not only accumulates accident-situation data but also represents injury data based on a human body coordinate system in a standardized and multilayered way ^(17)^. The principle, methods, and explanation of the results of the BISS are presented in discussion. Statistical analyses of sex and the types of injuries were performed using the chi-squared test.

### Ethical approval

This observational study was approved on October 25, 2013 (U 006) by the review board of Seitoku University and those of participating institutions in accordance with the ethical guidelines and regulations of the Declaration of Helsinki. It is exceedingly difficult to obtain written consent from the parents whose children suffered from injuries, who may feel panicked or have a guilty conscience. The review boards of Seitoku University and of participating institutions allowed not to take written consent, but to obtain oral agreements from the parents to participate in our study.

## Results

### 1. Recovery of questionnaire

We sent a letter to nursery schools and kindergartens located in the Tokyo metropolitan area and four surrounding prefectures, where the students of Seitoku University undergo internship for practice. We sent 279 questionnaires to kindergartens and 595 to nursery schools, and the numbers of responses received were 28 for kindergartens (10.0%) and 23 for nursery schools (3.9%). Most of the facilities were private; only a few were public facilities.

### 2. Registered institutions

A total of 51 institutions (23 nursery schools and 28 kindergartens) were registered in group A, three in group B, and 9 in group C, which included Sagamihara Ryouikuen (for children with severe motor and intellectual disabilities), the nurse’s room for handicapped children in Kitasato University Hospital, and 7 schools for handicapped children in Chiba prefecture.

### 3. Study period and numbers of children registered

After ethical approval was obtained, the study started in January 2014 and ended at the end of November in 2015. The overall study period was from 2013 to 2015, which includes the 1 year required for research preparation and ethical approval. In all, 383 injury cases were registered during the study period, of which 198 cases were in group A, 144 were in group B, and 41 were in group C.

### 4. Characteristics of injuries in the three groups

#### 1) Group A (nursery school and kindergarten children)

##### (1) Sex difference

More males than females were injured in this group (M/F: 119/47, p < 0.05); however, there was no sex difference for those aged under 3 years (M/F: 48/47) (Table 1).

**Table 1.** Sex Difference of Incidence of Injury Cases between Nursery School and Kindergarten Children.

	Nursery school	Kindergarten	Total
Male	48	67	115
Female	47	36	83
Total	95	103	198

The incidence of injuries among males is higher than that among females in kindergarten children and the total (p < 0.01, p < 0.05)

##### (2) Times when injuries occurred

**Figure 1.**
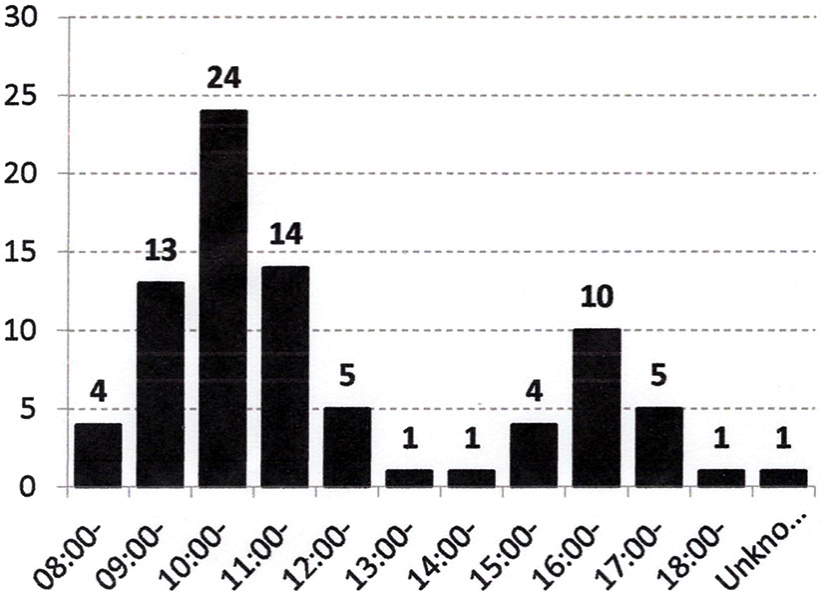
There was a clear biphasic pattern in nursery school children, in whom injuries occurred at around 10 in the morning and 4 in the afternoon.

There was no special time or day of the week when injuries were more prevalent for children in kindergarten; however, there was a clear biphasic pattern in nursery schools, in which injuries occurred at around 10 in the morning and 4 in the afternoon (Figure 1).

##### (3) Causes and types of injuries in group A

**Figure 2.**
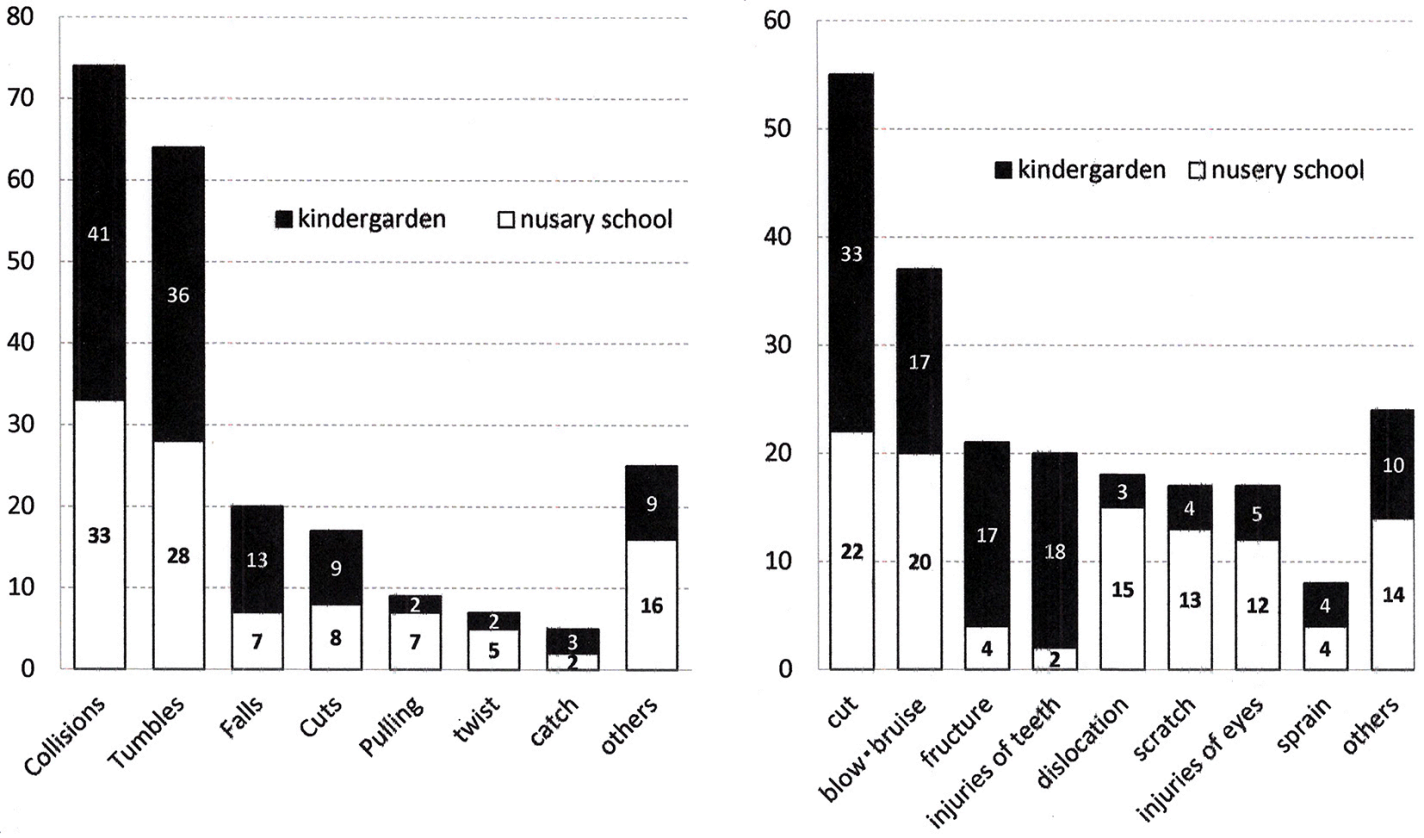
The causes (left) and types (right) of injuries in nursery school and kindergarten children (group A). Fractures and injuries of teeth occurred in kindergarten children and dislocations occurred in nursery school children.

The causes of injuries were, in order of frequency, collisions, falls, cutting, and pulling, and the types of injuries were cuts, bruises, abrasions, fractures, dislocations, sprains, and injuries of teeth (Figure 2). There were striking differences in the types of injuries of nursery school children (aged <3 years) and those in kindergarten (aged >3 years), with fractures and injuries of teeth occurring in kindergarten children (p < 0.01) and dislocations, with most cases of pulled elbows, occurring in nursery school children (p < 0.01) (Figure 2).

#### 2) Group B

##### (1) Sex difference

More males than females were injured in this group (M/F: 105/39, p < 0.01). The age distribution of injuries was as follows: the maximum number of injuries was observed at 7 years of age, followed by those at 11 years of age. At age more than 12 years, the injuries decreased. The lower grade students at primary school have a high peak.

##### (2) Causes of injuries

**Figure 3.**
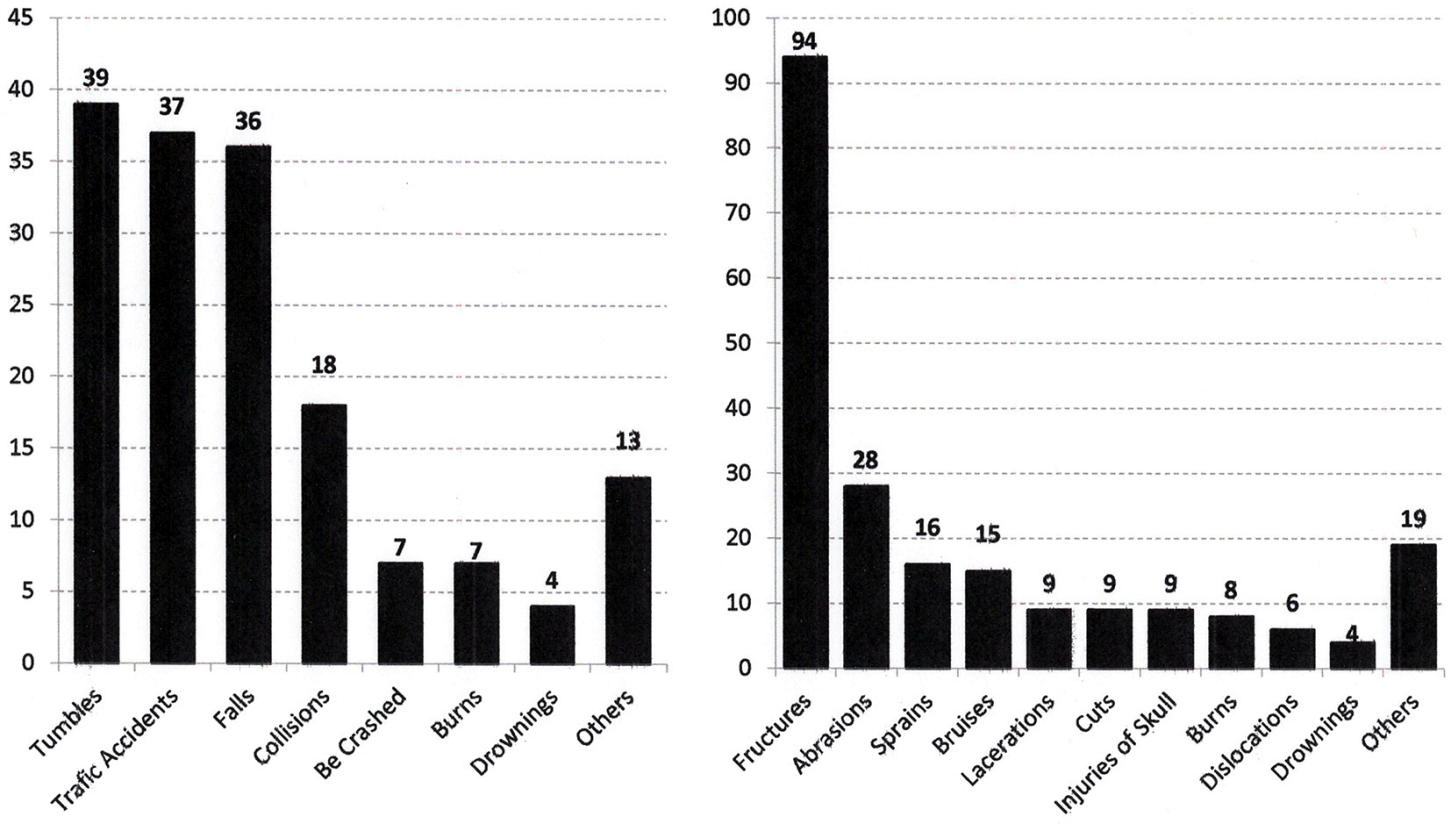
The causes (left) and types (right) of injuries in group B. The traffic accidents by bicycles and fractures were the most prominent types of injuries in this group.

The most common causes of injuries that occurred were, in order of frequency, falls, traffic accidents, collisions, and near drowning (Figure 3). Among the traffic accidents (37 cases), 20 cases were bicycle accidents, 12 children were hit by cars when walking, and 5 were due to other reasons such as using a scooter or balance bike.

##### (3) Types of injuries

The types of injuries were, in order of frequency, fractures, abrasions, sprains, bruises, and lacerations (Figure 3). Fractures accounted for 65.3% of all injuries in this group.

#### 2) Group C

##### (1) Sex difference

More males than females were injured in group C (M/F: 30/11, p < 0.01). The ages of the subjects ranged from 2 to 18 years. Although the age distribution was more like group B, the types of injuries more closely resembled those of group A. The children in this group suffered mainly from autism spectrum disorder (ASD) and attention-deficit hyperactivity disorder (ADHD). The intellectual levels of half of these children were normal or borderline.

##### (2) Causes of injuries

**Figure 4.**
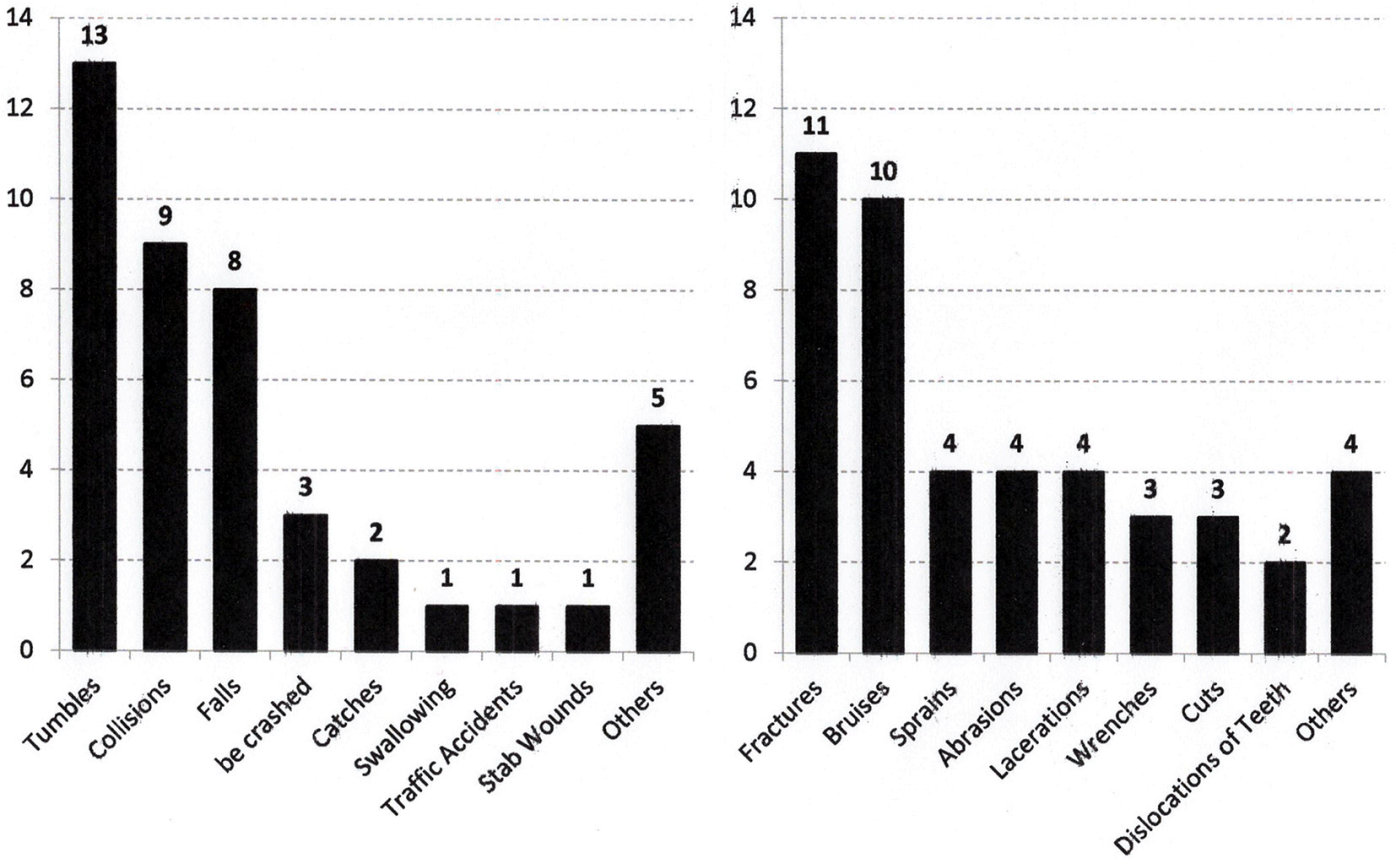
The causes (left) and types (right) of injuries in group C. The characteristic feature of this group was that injuries occurred suddenly and repeated cases were observed.

The causes of injuries were, in order of frequency, falls and collisions, and the types of injuries were fractures, bruises, sprains, and lacerations (Figure 4). The characteristic feature of this group was that injuries occurred suddenly and that repeated cases were observed.

##### (3) Types of injuries

The main types of injuries in this group were fractures and bruises, followed by sprains, abrasions, and cuts (Figure 4).

### 5. Bodygraphic Injury Surveillance System (BISS) analyses

**Figure 5.**
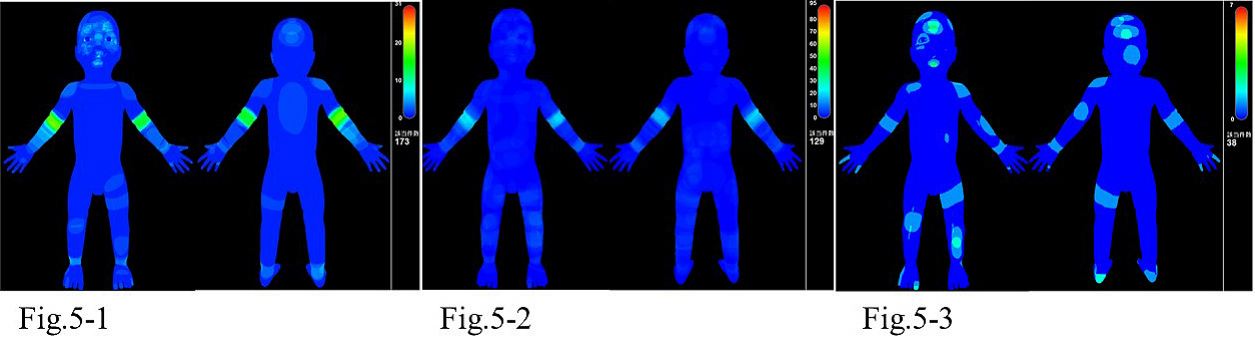
The Bodygraphic Injury Surveillance System (BISS) analysis in group A (Figure 5-1), group B (Figure 5-2), and group C (Figure 5-3). The injuries to the head were prominent in groups A and C, whereas those of the knee and ankle joints were prominent in group B.

Injuries to the head were prominent in groups A and C, whereas those of the knee and ankle joints were prominent in group B (Figure 5).

## Discussion

In this paper, we report the causes and types of injuries of 383 children. This study was not population based and did not estimate the incidence of child injuries in Japanese children, as reported previously for the city of Oomura ^(10)^ and in other counties ^(2), (16), (18), (19)^. The incidence of childhood injuries differed depending on the definition or severity of the injury and the age of the subjects. In Oomura, 20,300 individuals were aged under 19 years and among them, 1,728 cases of injuries were reported from March 2010 to July 2017. The annual incidence was 1,024/100,000 ^(10)^. In California, injury cases were surveyed every 3 months from 1996 to 1998 and 23,173 cases were collected. The peak incidence occurred between 15 and 17 months of age, and the annual incidence was 371/100,000 ^(19)^.

We collected information on 383 children from 3 sources. In group A, which consisted of nursery school (aged <3 years; 95 cases) and kindergarten children (aged 3-6 years; 108 cases), we found striking differences in sex and the types of injuries between the two age groups. As reported in the literature, males had more childhood injuries ^(2), (11), (13), (14), (16)^. Our results also showed male predominance in 198 cases of group A (M/F: 115/83). However, when we divided group A by age, that is, those aged less than 3 years and those 3 years and older, the sex difference disappeared in the younger group (M/F: 48/47). It is believed that the behavior of males is controlled by the exposure of the central nervous system to androgens during the fetal period ^(20)^. However, the actions and movements of children under 3 years are not as vigorous or likely to cause injuries as those of older children. Male-dominant injury cases have been reported from Japan ^(11)^ as well as other countries ^(2), (13), (14), (16)^, although these reports did not compare very young and older children.

Injuries of nursery school children occurred primarily at 2 times: 10 AM and 4 PM. Most nursery school children take a nap during the daytime after lunch, and this might be the reason for the biphasic times of injuries. In kindergarten children, such a phenomenon was not observed. In the report from Oomura where all injury cases are registered, the same phenomenon was observed ^(10)^.

The causes of injuries in group A were, in order of frequency, crashes, falls, and collisions. There was a striking difference in the types of injuries between nursery school and kindergarten children. Fractures and injuries of the teeth occurred in kindergarten children as a result of falling from jungle gyms or collisions with each other in the playground. On the other hand, among nursery school children, dislocations and abrasions were predominant. As they move together, holding hands, when someone falls suddenly, the hands are strongly pulled, which is one of the main causes of dislocation in young children in nursery schools. No previous report pointed out the difference between the incidences of fractures and dislocations during early childhood

In group B, most of the children were primary and junior high school students, and the causes of their injuries were traffic accidents, falling, burns, etc. Among the traffic accidents, 47% were bicycle accidents, 38 were injured while walking or riding in a car, and the others occurred while using a scooter or balance bike, or other factors. Traffic accidents, particularly bicycle accidents, were the most common in this age group. The rate of bicycle possession among children in Oomura is the highest in Japan ^(21)^. The prevention of bicycle accidents is particularly important in these age groups. The city of Oomura has conducted systemic education for the safe use of bicycles for the past 3 years, including education at school about the importance of wearing a helmet, proper adjustment of brake levers, etc. As a result, the incidence of bicycle accidents decreased from 90 cases per year to 45 cases per year ^(21)^.

In group C, although the children’s ages ranged from 5 to 15 years, which was close to that of group B, the causes of injuries were collisions, falls, being crushed, etc., which were more like those of group A. A characteristic feature of children with ASD and ADHD is sudden and unexpected movement, which causes injuries. Children with disability have characteristic features of dyskinesia, impulsiveness, and disorders of integration of the senses and body movements ^(22), (23), (24)^.

The BISS is a new technology developed by the AIST. Head injuries were the most prominent in groups A and C (Figure 5-1 and 5-3) and injuries of the extremities were the most prominent in groups B and C (Figure 5-2 and 5-3). The BISS allows us to input information by typing in text data or outlining on a three-dimensional human body model with a computer mouse; then, the input data are digitized into a raster model and relevant information can be obtained. All input data are stored in a database for retrieval, analyses, and visualization, according to need.

**Figure 6.**
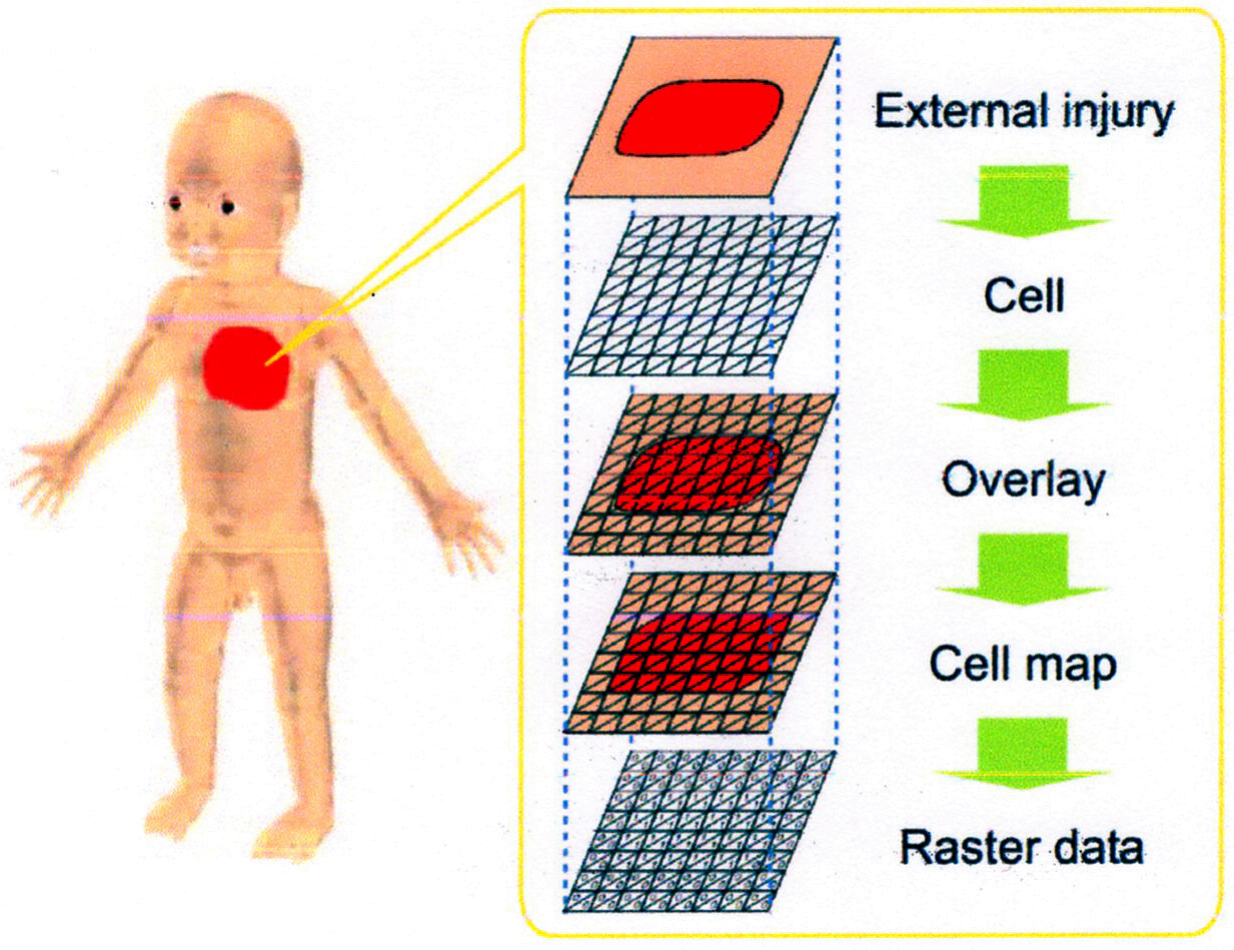
Representation model of an injury using raster data.

Injury information can be classified into three types: location, shape, and attribute. In the BISS, this information is raster data that are structured as multilayers based on the location on the human body, as shown in Figure 6. In this paper, raster data indicate an injury as a set of cells sectioned on the body surface. Each cell has a coordinate value in the human body coordinate system. Figure 6 shows input injury data converted to raster data. The injury data are overlaid on cells of the body surface and converted into a cell map. Each cell has information on the type of injury and the degree of severity, along with the coordinates in the human body coordinate system.

The BISS has the following functions: (1) input/output function; (2) information retrieval function; and (3) the International Statistical Classification of Diseases and Related Health Problems (ICD-10) Code Conversion function. For function (1), using the BISS, the shape data of an external injury is drawn on the three-dimensional human body model with a computer mouse and text data are typed in with a keyboard. The input injury data are converted to raster data and stored in the database system. As the standard human body model is used, injury data are normalized. For function (2), the BISS can retrieve target injury data by a spatial query and a text query and show the visual results. For example, by giving “1 year old” and “scald” as text queries, the BISS retrieves all scald data of 1-year-old children. By drawing a spatial query with a computer mouse, the BISS retrieves all injury data of the target body part. For function (3), the ICD-10, established by the World Health Organization, is an international statistical standard of the causes of death and disease. ICD-10 codes include items concerning injuries due to external causes; these items are defined by both the type of injury and the injured body part. ICD-10 codes are used widely in medical institutions worldwide.

Figure 5 shows the injury frequencies visualized by the BISS. As each instance of injury data is expressed in a normalized and structured form in the BISS, we can superimpose data and count frequencies by summing the data. The color bar in Figure 5 indicates the injury frequency. In the figures, the red color indicates the area with the highest frequency of external injury.

As shown in Figure 5, bodygraphic statistical analysis and childhood modeling using the BISS are tools used not only to accumulate accident-situation data but also to represent injury data based on a human body coordinate system in a standardized and multilayered way, to clarify the mechanisms of injury; then, will be reach to preventing injuries in future.

We reported 383 cases of childhood injuries from 3 sources for very young children, primary and junior high school students, and children with disability. Our results suggest that the BISS may help to clarify the mechanisms of injury and then reach to prevention of severe injuries in future.

## Article Information

### Conflicts of Interest

None

### Sources of Funding

This work was supported by a Grant-in-aid for Scientific Research from the Ministry of Education, Science, and Culture of Japan (B) 25293120).

### Acknowledgement

The authors thank the many nursery school and kindergarten teachers, and the staff of the hospitals and school for handicapped children who assisted us in this study. The authors also thank Michiyo Kanda for her help with the arrangement of the questionnaires.

### Author Contributions

N.M. is the first author and designed the study and wrote the manuscript. Y.N. and K.D. provided technical support of the BISS and performed the population-based Oomura study. S.H., K.T., and K.K. performed data collection in group A. S.K. performed data collection from Kitasato University Hospital, U.H. from Seitoku Primary School, and T.O. from Sapporo Tokusyuukai Hospital. N.H. performed data collection in group C.

### Approval by Institutional Review Board (IRB)

1. The institutional review board of Seitoku University No. 527 on October 25, 2013 (H25U006)

2. The institutional review board of Tokusyukai Hospital on March 20, 2014

3. The institutional review board of Kitasato University School of Medicine on October 9, 2013 (B-13-103)

4. The institutional review board of Sagamihara-Ryouikuenn on January 20, 2014
